# P-1570. D-mannose for Prevention of Recurrent Urinary Tract Infection in Adult Women: An Updated Systematic Review and Meta-analysis

**DOI:** 10.1093/ofid/ofae631.1737

**Published:** 2025-01-29

**Authors:** Mrinal Murali Krishna, Meghna Joseph, Chidubem Ezenna, Lal Sadasivan Sreemathy

**Affiliations:** Medical College Thiruvananthapuram, Mavelikara, Kerala, India; Medical College Thiruvananthapuram, Mavelikara, Kerala, India; UMass-Baystate medical center, Springfield, Massachusetts; ReAct Asia-Pacific, Global Institute of Public Health, Thiruvananthapuram, Kerala, India

## Abstract

**Background:**

Urinary tract infections (UTIs) are prevalent, especially among women as approximately 50% of all healthy women will encounter at least one episode of UTI during their lifetime. An estimated 25%-35% of them will experience a recurrence within the following year. Prolonged antibiotic therapy for prophylaxis of UTI poses the risk of antimicrobial resistance, alteration of normal flora, and high costs to the patient. D-mannose, a polysaccharide may reduce UTI by preventing the adherence of bacteria to uroepithelium by binding to the type1 pili and saturating the adhesin FimH. D-mannose is commonly marketed for urinary tract health and is touted to contribute to better antimicrobial management within primary care settings. We performed an updated meta-analysis comparing prophylaxis with D-mannose versus placebo or no treatment for prevention of recurrent UTI in adult women.

**Figure:**
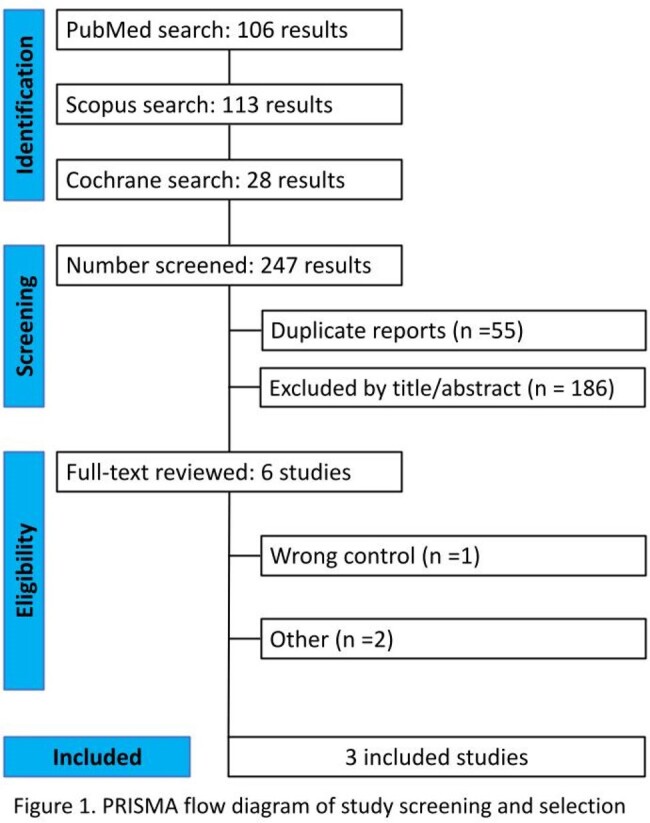

**Methods:**

We systematically searched PubMed, Scopus, and Cochrane Central databases for trials comparing D-mannose versus placebo or no treatment for UTI prophylaxis in adult women. Outcomes of interest included one or more episodes of UTI within 6 months of randomization and adverse events. Statistical analysis was performed using R software. Heterogeneity was assessed using I^2^ statistics. The analysis was conducted following the Preferred Reporting Items for Systematic Reviews and Meta-Analysis (PRISMA) guideline (Figure 1).

**Figure:**
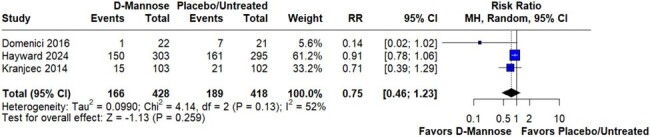

Risk estimation for recurrent UTI

**Results:**

The systematic review identified three randomized controlled trials including 846 participants. Prophylaxis with D-mannose was given to 428 (50.59%) participants. The risk of recurrent UTI within 6 months of randomization (RR 0.75; 95%CI 0.46-1.23; p=0.259; I^2^=52%) showed no significant difference between the groups (Figure 2). Adverse events (RR 0.81; 95%CI 0.09-6.91; p=0.847; I^2^=94%) were also similar between the groups (Figure 3).

**Figure:**
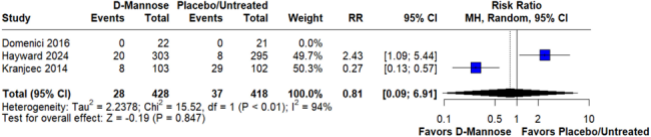

Risk estimation for adverse events

**Conclusion:**

In contrast to a previous meta-analysis, our study found that prophylaxis with D-mannose did not reduce the risk of recurrent UTIs in adult women. Adverse events were also comparable between the groups.

**Disclosures:**

**All Authors**: No reported disclosures

